# Uncovering the Cardioprotective Potential of Diacerein in Doxorubicin Cardiotoxicity: Mitigating Ferritinophagy-Mediated Ferroptosis via Upregulating NRF2/SLC7A11/GPX4 Axis

**DOI:** 10.3390/antiox13040493

**Published:** 2024-04-20

**Authors:** Rehab M. El-Gohary, Asmaa H. Okasha, Alaa H. Abd El-Azeem, Muhammad T. Abdel Ghafar, Sarah Ibrahim, Islam I. Hegab, Eman E. Farghal, Soha Abdel Fattah Shalaby, Ola A. Elshora, Aisha E. ElMehy, Amany Nagy Barakat, Basma Saed Amer, Fatma G. Sobeeh, Gehan H. AboEl-Magd, Asmaa A. Ghalwash

**Affiliations:** 1 Medical Biochemistry Department, Faculty of Medicine, Tanta University, Tanta 31511, Egypt; asmaa.hamdy@med.tanta.edu.eg (A.H.O.); asmaa.ghalwash@med.tanta.edu.eg (A.A.G.); 2 Medical Pharmacology Department, Faculty of Medicine, Tanta University, Tanta 31511, Egypt; alaa.hussien@med.tanta.edu.eg; 3 Clinical Pathology Department, Faculty of Medicine, Tanta University, Tanta 31511, Egypt; eman.farghal@med.tanta.edu.eg (E.E.F.); ola.elshoura@med.tanta.edu.eg (O.A.E.); 4 Human Anatomy and Embryology Department, Faculty of Medicine, Tanta University, Tanta 31511, Egypt; sara.ibrahim@med.tanta.edu.eg; 5 Medical Physiology Department, Faculty of Medicine, Tanta University, Tanta 31511, Egypt; islam.hegab@med.tanta.edu.eg; 6 Department of Bio-Physiology, Ibn Sina National College for Medical Studies, Jeddah 22413, Saudi Arabia; 7 Internal Medicine Department, Faculty of Medicine, Tanta University, Tanta 31511, Egypt; soha.shalaby@med.tanta.edu.eg; 8 Forensic Medicine & Clinical Toxicology Department, Faculty of Medicine, Tanta University, Tanta 31511, Egypt; aisha.elmihy@med.tanta.edu.eg (A.E.E.); fatma.sobeeh@med.tanta.edu.eg (F.G.S.); 9 Pediatric Department, Faculty of Medicine, Tanta University, Tanta 31511, Egypt; amany.barakat@med.tanta.edu.eg; 10 Pathology Department, Faculty of Medicine, Tanta University, Tanta 31511, Egypt; basma.amer@med.tanta.edu.eg; 11 Chest Diseases Department, Faculty of Medicine, Tanta University, Tanta 31511, Egypt; gehan.abouelmagd@med.tanta.edu.eg

**Keywords:** doxorubicin cardiotoxicity, iron, nuclear receptor coactivator 4-dependent ferritinophagy, ferroptosis, ferritin heavy chain polypeptide 1, oxidative stress, nuclear factor E2-related factor 2/solute carrier family 7 member 11/ glutathione peroxidase 4 axis, diacerein

## Abstract

Doxorubicin (DOX)-induced cardiotoxicity (DIC) is a life-threatening clinical issue with limited preventive approaches, posing a substantial challenge to cancer survivors. The anthraquinone diacerein (DCN) exhibits significant anti-inflammatory, anti-proliferative, and antioxidant actions. Its beneficial effects on DIC have yet to be clarified. Therefore, this study investigated DCN’s cardioprotective potency and its conceivable molecular targets against DIC. Twenty-eight Wister rats were assigned to CON, DOX, DCN-L/DOX, and DCN-H/DOX groups. Serum cardiac damage indices, iron assay, oxidative stress, inflammation, endoplasmic reticulum (ER) stress, apoptosis, ferritinophagy, and ferroptosis-related biomarkers were estimated. Nuclear factor E2-related factor 2 (NRF2) DNA-binding activity and phospho-p53 immunoreactivity were assessed. DCN administration effectively ameliorated DOX-induced cardiac cytomorphological abnormalities. Additionally, DCN profoundly combated the DOX-induced labile iron pool expansion alongside its consequent lethal lipid peroxide overproduction, whereas it counteracted ferritinophagy and enhanced iron storage. Indeed, DCN valuably reinforced the cardiomyocytes’ resistance to ferroptosis, mainly by restoring the NRF2/solute carrier family 7 member 11 (SLC7A11)/glutathione peroxidase 4 (GPX4) signaling axis. Furthermore, DCN abrogated the cardiac oxidative damage, inflammatory response, ER stress, and cardiomyocyte apoptosis elicited by DOX. In conclusion, for the first time, our findings validated DCN’s cardioprotective potency against DIC based on its antioxidant, anti-inflammatory, anti-ferroptotic, and anti-apoptotic imprint, chiefly mediated by the NRF2/SLC7A11/GPX4 axis. Accordingly, DCN could represent a promising therapeutic avenue for patients under DOX-dependent chemotherapy.

## 1. Introduction

Doxorubicin (DOX) is an effective chemotherapeutic agent for treating malignant tumors, but its clinical application results in irreversible, devastating, and possibly life-threatening cardiotoxicity [1]. DOX primarily provokes cardiac dysfunction and heart failure by augmenting pro-inflammatory cytokines, oxidative stress (OS) products, pro-apoptotic mediators, and autophagy biomarkers [2]. Even after decades of investigation, pharmacological interventions remain inadequate [about one-fourth of patients experience DOX-induced cardiotoxicity (DIC)]; this may be attributed to the highly sophisticated molecular pathways implicated in DIC [3]. Thus, it has become imperative to introduce new effective adjuvants to enhance DOX tolerability without interference with its anti-neoplastic efficacy.

Recent evidence suggests that ferritinophagy-mediated ferroptosis is one of the newly discovered molecular pathways implicated in the pathophysiology of various cardiac illnesses, notably DIC [3,4]. Ferroptosis is a novel oxidative non-apoptotic type of regulated cell death marked by iron-based accumulation of toxic levels of reactive oxygen species (ROS) and augmenting oxidative damage to lipids, proteins, and DNA in numerous cell types, including cardiomyocytes, inducing cytotoxic effects [1,5]. Mechanistically, DOX upregulated nuclear receptor coactivator 4 (NCOA4) expression, which mediates selective autophagic/lysosomal degradation of ferritin, defined as ferritinophagy, subsequently releasing an enormous amount of redox-active iron [4]. Biochemically, an excessive accumulation of ferrous iron (Fe^2+^) within the cell triggers ferroptosis by ROS overproduction via Fenton’s reaction, resulting in reduced glutathione (GSH) depletion, glutathione peroxidase 4 (GPX4) dysfunction, and lipid peroxidation, ultimately ending in membrane fragmentation and cell death [6]. Therefore, pharmacologically targeting ferritinophagy/ferroptosis may be a novel interventional modality for counteracting DIC and restoring cardiac function in patients receiving DOX chemotherapy.

Nuclear factor E2-related factor 2 (NRF2) is an antioxidant response core and a cell survival mediator [7] that regulates crucial cellular defense mechanisms and protects against cardiac damage [8]. A recent study supported its anti-ferroptotic imprint via modulating solute carrier family 7 member 11 (SLC7A11) [7]. SLC7A11, a cystine/glutamate antiporter, contributes to GSH biosynthesis and defends cells from OS and ferroptotic death [9]. Further, many proteins that are tightly associated with ferroptosis, such as GPX4 and ferritin heavy polypeptide 1 (FTH1), are established NRF2 target genes [10]. Accordingly, NRF2 might be a novel target for future ferroptosis-dependent DIC therapy.

Diacerein (DCN) is an anthraquinone derivative with prominent analgesic and anti-inflammatory properties approved for osteoarthritis management [11]. DCN’s primary molecular mechanism is based on suppressing interleukin 1 beta (IL-1β) and its downstream signaling, including NF-κB activation [12]. Based on its anti-inflammatory, antioxidant, antiproliferative, and anti-apoptotic activities, DCN has proven effective in treating several diseases in human and animal studies [13,14]. DCN and rhein, its active metabolite, recently possess anti-neoplastic and chemo-sensitization effects [15]. Moreover, DCN can be applied as an adjuvant to chemotherapy to counteract adverse effects, enhance tolerability, and diminish multidrug resistance [16,17].

Nevertheless, to the best of our knowledge, no studies have investigated DCN’s cardioprotective effects on DIC. Therefore, our objectives were to assess the potential cardioprotective effects of DCN against cardiac dysfunction and cardiomyocyte injury triggered by DOX. Furthermore, we aimed to gain mechanistic insights into the signaling cascades involved in this protective role, highlighting the potential anti-ferroptosis activity of DCN in cardiomyocytes as a novel molecular mechanism.

## 2. Materials and Methods

### 2.1. Drugs and Chemicals

DCN was obtained from Eva Pharma Company, Egypt, and was prepared immediately before its use by dissolving in the vehicle. Active pharmaceutical ingredients of DOX (CAS: 25316-40-9) and other chemicals used were purchased from Sigma Chemicals Co. (St. Louis, MO, USA). All chemicals and solvents were of high analytic grade.

### 2.2. Experimental Design

**Figure 1:**
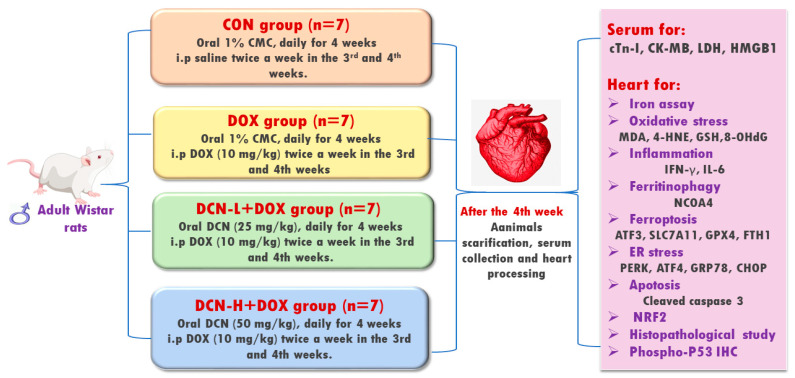
Schematic representation of the study protocol.

Twenty-eight adult male 6–8-week-old Wistar albino rats, weighing 170 ± 40 g, were randomly allocated into four equal groups (Figure 1). Group I (CON) received 1% carboxymethylcellulose (vehicle) daily by oral gavage for four weeks and intraperitoneal injection (i.p.) of saline twice a week in the 3rd and 4th weeks. Group II (DOX) received the vehicle by oral gavage for four weeks and i.p. of DOX (10 mg/kg) twice a week in the 3rd and 4th weeks [18]. Group III (DCN-L+DOX) received DCN (25 mg/kg) daily by oral gavage for 4 weeks with concomitant i.p. DOX injection (10 mg/kg) twice a week in the 3rd and 4th weeks. Group IV (DCN-H+DOX) received DCN (50 mg/kg) daily by oral gavage for 4 weeks with concomitant i.p. DOX injection (10 mg/kg) twice a week in the 3rd and 4th weeks. The dosage of DCN, at 25 and 50 mg/kg/day, has been determined based on a prior study demonstrating its tissue-protective effects [19]. The animal study protocol was approved by the Medical Research Ethics Committee, Faculty of Medicine, Tanta University (code: 36264PR266/7/23).

### 2.3. Sampling

#### 2.3.1. Blood Sampling and Serum Preparation

At the end of the abovementioned period and overnight fasting, rats were anesthetized using diethyl ether. Blood samples were withdrawn while the hearts were beating through the intracardiac puncture, followed by cervical decapitation. Blood samples were immediately collected into dry sterile centrifugation tubes, and then centrifuged for 20 min (1000× *g*/4 °C). Sera were collected and stored at −80 °C for further biochemical investigation.

#### 2.3.2. Collection of Heart and Calculating Heart-to-Body Weight (HW/BW) Ratio

After the collection of blood samples, the thoracic cages were opened, and the cardiac tissues were dissected, carefully washed with ice-cold saline to remove extraneous materials, and blotted individually on filter paper. They were weighed (mg) and divided by the total body weight in grams to calculate the HW/BW ratio. Next, each rat’s cardiac tissue was divided into four specimens. Both atria were homogenized in phosphate-buffered saline (50 mM, pH 7.4), containing a proteinase inhibitor, and centrifuged at 5000 rpm for 20 min at 4 °C. The resultant supernatants were kept at −80 °C until the biochemical assay, and their protein contents were determined according to the Bradford technique [20]. Meanwhile, half of the left ventricle was wrapped in aluminum foil and stored at −80 °C until use for molecular analysis, while the other half was subjected to nuclear protein extraction using the nuclear protein extraction kit (Biobasic Inc., Markham, ON, Canada; Cat# BSP002) following the manufacturer’s protocol. Principally, it involves the addition of cytoplasmic protein extraction solution to the sample, providing a hypotonic condition, breaking the cell membrane, and releasing the proteins, followed by centrifugation for the collection of the nucleoli (deposit). Then, the nuclear proteins were extracted by adding a nuclear protein extraction solution, followed by centrifugation. An additional protease inhibitor cocktail and phosphatase inhibitor cocktail were included to maintain protein integrity and high activity. In addition, dithiothreitol (DTT) helps to maintain the reduced state of the environment by avoiding the false interaction between the cysteines, so the protein will stay pure. The resultant supernatants were kept at −80 °C for NRF2 DNA-binding activity (Abcam Co., Cambridge, UK; Cat# ab207223) and CHOP assays (MyBioSource Co., San Diego, CA, USA; Cat# MBS3808179). On the other hand, the right ventricle of the cardiac tissue was preserved in a 4% paraformaldehyde solution before being dried and embedded in paraffin for histopathological and immunohistochemical assays.

### 2.4. Biochemical Assay

#### 2.4.1. Assay of Cardiac Toxicity Biomarkers

Serum levels of cardiac troponin (cTn-I) were assessed by the Cusabio ELISA Kit (Cusabio, Wuhan, China; Cat# CSB-E08594r) according to the manufacturer’s protocol, whereas serum creatine kinase isoenzyme-MB activity was assessed using a commercially available CK-MB assay kit (Sigma-Aldrich, Tokyo, Japan; Cat# MAK116), and the results were expressed as U/L. Meanwhile, serum lactate dehydrogenase (LDH) activity was assayed using a commercial colorimetric kit (BioVision, CA, USA; Cat# K726-500), where LDH reduces NAD+ to NADH and interacts with a probe to produce a reduced chromogen whose optical density was measured at 450 nm.

#### 2.4.2. Determination of Cardiac Activating Transcription Factor (ATF) 3 and Fe^2+^

ATF3 levels were immunoassayed in cardiac tissue homogenate using a rat commercial ELISA kit (MyBioSource Co., San Diego, CA, USA; Cat# MBS041276). Cardiac Fe^2+^ was calorimetrically measured using a commercial ferrous iron assay kit (Cat# E-BC-K304-S) supplied by Elabscience Biotechnology Co., Houston, TX, USA, where Fe^2+^ is released by the addition of the acidic buffer and reacted with a chromogen (bipyridine), resulting in a colorimetric (520 nm) product proportional to the iron concentrations in the sample.

#### 2.4.3. Detection of Cardiac Redox Status Biomarkers

To assess cardiac lipid peroxidation, malondialdehyde (MDA) and 4-hydroxynonenal (4-HNE) levels were assessed in cardiac tissue homogenates. MDA was colorimetrically assayed by a thiobarbituric acid (TBA)-dependent method where thiobarbituric acid reactive substances, a pink-colored product of a 30-min TBA reaction with MDA in an acidic medium with a temperature of 95 °C, were measured at 534 nm. This method was conducted according to Mihara and Uchiyama’s protocol [21] using a specific Bio-diagnostic Kit (Cat# MD2529; Giza, Egypt). Meanwhile, 4-hydroxynonenal (4-HNE) and 8-hydroxy-2′-deoxyguanosine (8-OHdG), a marker of oxidative DNA damage, levels were immunoassayed in cardiac tissue homogenate using MyBioSource company rat ELISA kits (Cat# MBS8807298 and MBS269902, respectively), while the procedures were sticky to the manufacturer’s guidelines. As for the cardiac antioxidant status, it was assessed by determining GSH level and GPX4 activity. Cardiac GSH level was colorimetrically determined using a Bio-diagnostic commercial kit (Cat# GR2511; Giza, Egypt). The rationale for this kit is the GSH-dependent reduction of 5,5′-Dithiobis (2-nitrobenzoic acid), yielding a yellow reduced chromogen directly proportional to GSH concentration, according to Ellman’s protocol [22]. As per the manufacturer’s instructions, a volume of 0.5 mL of cardiac homogenate supernatant (dilution range: 5–10 in cold phosphate buffer, 50 mM) was added to 0.5 mL of trichloroacetic acid (500 mM), incubated for 5 min at room temperature, then centrifuged for 15 min at 3000 rpm, and the supernatant was obtained. Then, a volume of 0.5 mL of the supernatant was added to 1 mL of phosphate buffer 100 mM and 0.1 mL of 5,5′dithiobis (2-nitrobenzoic acid) 1 mM and mixed well. The absorbance was measured after 10 min against a blank at 405 nm using a semiautomatic BTS-350 Biosystems spectrophotometer. The GPX4 activity was also assessed using a commercial kit obtained from Bio-diagnostic Co., Giza, Egypt. The assay is an indirect measure of GPX4 activity where oxidized glutathione is produced upon reduction in an organic peroxide by GPX4, according to the Paglia and Valentine protocol [23].

#### 2.4.4. Evaluation of Inflammation-Related Biomarkers

Cardiac levels of interferon-gamma (IFN-γ) were assayed by using an INVITROGEN ELISA Kit (Cat# BMS621; Bender MedSystems, Vienna Biocenter, Austria). Besides, cardiac tissue levels of IL-6 and serum levels of high mobility group box 1 (HMGB1) were immunoassayed by rat commercial ELISA kits purchased from MyBioSource Company (San Diego, CA, USA; Cat# MBS269892 and MBS703437, respectively).

#### 2.4.5. Assay of Cardiac NRF2 DNA-Binding Activity, ER Stress Sensors, and Cleaved Caspase-3

Regarding cardiac NRF2 DNA-binding activity and C/EBP homologous protein (CHOP), a preliminary step of nuclear protein extraction was performed using a nuclear extraction kit, followed by determining their levels in the nuclear extract using the corresponding ELISA kits. The results were adjusted for tissue proteins. Cardiac ATF4, glucose-regulated protein 78 (GRP78), and cleaved caspase-3 levels were immunoassayed in cardiac tissue homogenate using rat commercial ELISA kits acquired from MyBioSource Co. (Cat# MBS2024531, MBS035991, and MBS261814, respectively).

### 2.5. Molecular Assay of NCOA4, FTH1, SLC7A11, and Protein Kinase-Like ER Kinase (PERK) by Quantitative RT-PCR Analysis

Total RNA was isolated from cardiac samples using the Gene JET RNA Purification Kit (Thermo Scientific, Waltham, MA, USA; Cat# K0731). After being quantified by Nanodrop to assess the purity and quantity, total RNA (5 μg) was then reverse transcribed to produce cDNA using Revert Aid H Minus Reverse Transcriptase (Thermo Scientific, Waltham, MA, USA; Cat# EP0451). The cDNA was used as a template to determine the relative expression of the NCOA4, FTH1, SLC7A11, and PERK genes using the Step One Plus real-time PCR system (Applied Biosystems, Foster, CA, USA). The sequences of the applied primers are shown in Table 1. The relative gene expression was determined using the 2^−ΔΔCt^ method [24].

### 2.6. Histopathological Examination

Paraformaldehyde-preserved cardiac tissues were processed in paraffin, and 5-µm-thick paraffin sections were stained with hematoxylin and eosin (H&E) to assess cardiac tissue histopathological changes. The stained sections were microscopically examined at different magnification powers using a light microscope (Olympus, Tokyo, Japan). Other sections were stained with MASSON solutions to detect the cardiomyocyte’s collagen content [25]. According to previous descriptions [26], the degree of cardiac myocyte degeneration in the heart section was evaluated on a scale of 0–3. A score of 0 was given when there was no evidence of myofibrillar degeneration. A score of 1 was assigned when less than 5% of cells exhibited early myofibrillar loss. A score of 2 was given when 15–30% of cells displayed significant myofibrillar loss and/or cytoplasmic degeneration. A score of 3 was assigned for diffuse damage exceeding 30%, with most cardiac myocytes exhibiting myofibrillar disruption and noticeable loss of contractile components.

### 2.7. Immunohistochemical Analysis

Immunostaining was performed on cardiac tissue in paraffin-embedded sections for the reactivity of phosphorylated p53. The paraffin blocks of cardiac tissues were heated in a 60 °C oven for 30 min, routinely deparaffinized, and hydrated using rabbit polyclonal antibodies specific for phosphorylated p53 (Thermo Fisher Scientific, Waltham, MA, USA; Ser15; 1:1000; Cat# PA5-104742) [25].

### 2.8. Morphometric Analysis

Morphometric analysis was conducted using image analysis tools. The quantification of immunohistochemistry and Masson’s trichrome images was performed using Image J software 1.46a (NIH, Bethesda, MD, USA). In ten non-overlapping regions of each section, the mean area percentage of collagen fibers and the positive immunoreaction of phospho-p53 were counted at (×400 magnification).

### 2.9. Statistical Analysis

For data expression, the mean and standard deviation were used. The Shapiro-Wilk test was used to assess the data’s normality. A one-way analysis of variance followed by the Tukey test was used to compare multiple groups. Pearson’s correlations were used to correlate NRF2 and other measured parameters in the DCN-H+DOX group. In addition, the Kruskal-Walls test and the Mann-Whitney *U* test were used for the statistical analysis of histopathological scores. The data were analyzed and graphed using GraphPad Prism 5.02. A *p*-value of less than 0.05 was considered statistically significant.

## 3. Results

### 3.1. DCN-Attenuated DOX-Induced Cardiomyocyte Injury

**Figure 2:**
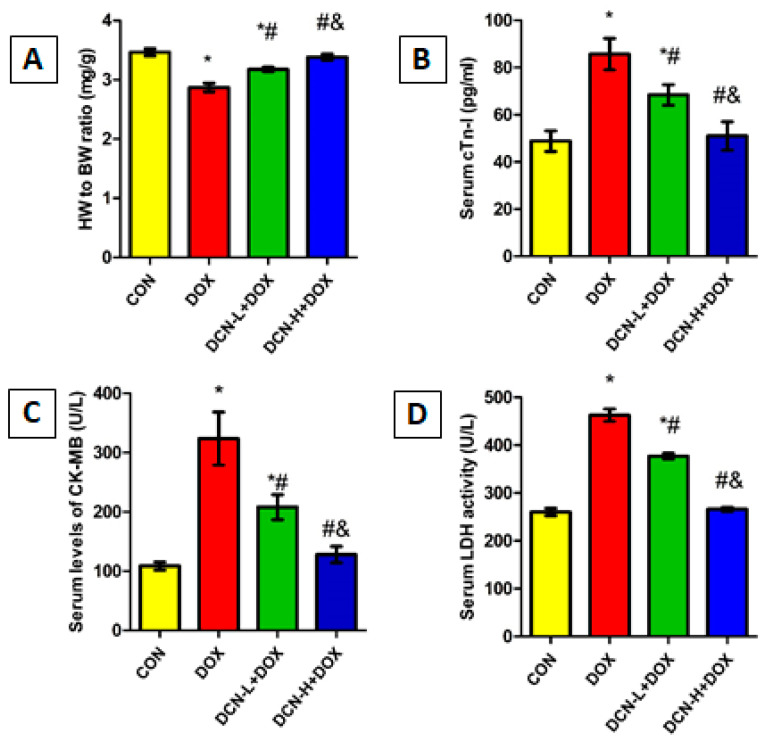
HW/BW ratio and cardiac toxicity indices among different studied groups. (**A**) HW/BW ratio (mg/g). (**B**) Serum cTn-I levels (pg/mL). (**C**) Serum CK-MB activity (U/L). (**D**) Serum LDH activity (U/L). CON: control; DOX: doxorubicin; DCN-L: diacerein low dose; DCN-H: diacerein high dose; HW: heart weight; BW: body weight; cTn-I: cardiac troponin-I; CK-MB: creatine kinase isoenzyme-MB; LDH: lactate dehydrogenase. *p* < 0.05, values expressed as mean ± SD (*n* = 7), * vs. CON group, # vs. DOX group, & vs. DCN-L+DOX group.

DOX-injected rats showed a marked reduction in HW/BW ratio with a substantial boost in cardiac toxicity indices, including cTn-I, CK-MB, and LDH, compared with the CON group, indicating myocardial injury. Meanwhile, DCN treatment significantly and dose-dependently improved the HW/BW ratio and reduced cardiac toxicity markers compared to the untreated DOX group (Figure 2).

### 3.2. DCN-Alleviated DOX-Provoked Cytomorphological Abnormalities in Heart Tissue

**Figure 3:**
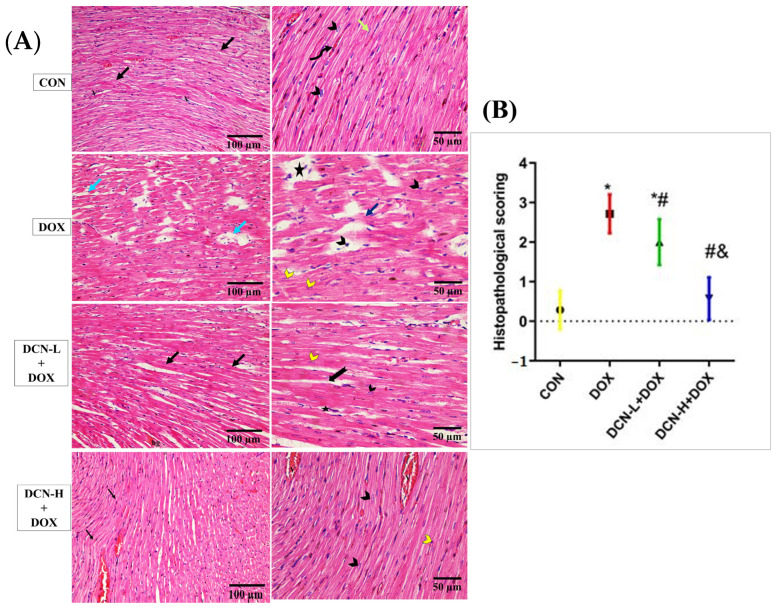
Modulatory effect of DCN on histopathological analysis of heart tissue. (**A**) In light microscopic images of all research groups, the CON group demonstrates normal cardiac muscle in the longitudinal section with a syncytium of branching and anastomosing myocardial fibers (black arrows). On higher magnification, the central nuclei (arrowhead), blood capillaries (V), and some lipofuscin granules are seen near the nuclei (curved arrow), with flat fibroblasts forming the interstitial space (green arrow). The (DOX) group demonstrates disorganized myocardial fibers with degenerative changes (blue arrow) in the form of focal disturbance of some myocardial fibers (dark blue arrow), interstitial edema in between the disrupted cardiomyocytes, and pyknotic and karyolitic nuclei (black and yellow arrowheads, respectively). Areas of sarcoplasmic vacuolation (star) are observed. The DCN-L+DOX group demonstrates some degree of enhancement as organized myocardial fibers (black arrow) with central vesicular nuclei (black arrowhead). However, the area of hemorrhage (hg), distribution of some fibers (bifid arrow), and karyolitic nuclei (yellow arrowhead) of some cardiomyocytes are still observed. The DCN-H+DOX group demonstrates apparent enhancement where they display normal acidophilic sarcoplasm (black arrow) and central vesicular nuclei (black arrowhead), but pyknotic nuclei in a few cells (yellow arrowhead) and congested blood capillaries (V) are still observed. (**B**) Histopathological score of heart injury, significant at *p*-value < 0.05. Values are expressed as mean ± SD, with 7 rats in each group. * Significant vs. CON group, # significant vs. DOX group, & significant vs. DCN-L+DOX group (H&E ×200, scale bar = 100 μm, H&E ×400, scale bar = 50 μm). CON: control; DOX: doxorubicin; DCN-L: diacerein low dose; DCN-H: diacerein high dose.

A light microscopic examination of H&E-stained sections of cardiac tissues in the CON group identified a normal histological architecture of the cardiac muscle. This architecture comprises a syncytium of branching and anastomosing myocardial fibers, central nuclei, and blood capillaries. Some lipofuscin granules are seen near the nuclei, with flat fibroblasts forming the interstitial space. Meanwhile, the DOX group demonstrated degenerative changes in the form of focal disturbance of some myocardial fibers, interstitial oedema between disrupted cardiomyocytes, pyknotic and karyolitic nuclei, and areas of sarcoplasmic vacuolation. On the contrary, the DCN-L+DOX and DCN-H+DOX groups demonstrated variable degrees of improvement, with more advanced improvement in the latter group than in the former. Compared to the DOX group, the estimation of the heart injury score indicated considerable improvement in the DCN-L+DOX and DCN-H+DOX groups, with the DCN-H+DOX group exhibiting the greatest improvement. The DCN-H+DOX group did not significantly differ from the CON group (Figure 3).

### 3.3. Modulatory Effects of DCN on Cardiac Collagen Fiber Content

**Figure 4:**
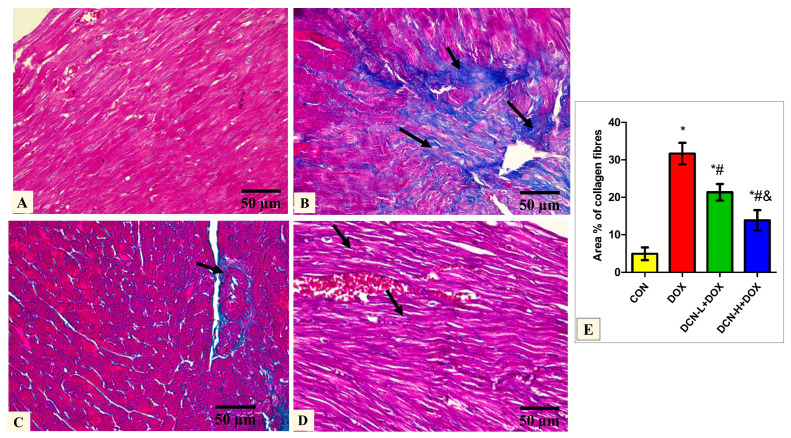
Modulatory effect of DCN on Masson’s trichrome staining heart tissue. (**A**) The CON group demonstrates sparse collagen fibers in between the cardiomyocytes. (**B**) The DOX group displays plentiful collagen fiber (arrows) deposition in between the cardiomyocytes. (**C**) The DCN-L+DOX group shows moderate collagen fibers (arrows) between cardiomyocytes and around the blood capillaries. (**D**) The DCN-H+DOX group demonstrates minimal collagen fibers (arrows) between the cardiomyocytes. (**E**) Area percentage of collagen fibers in all groups. (Masson’s trichrome stain ×400, scale bar = 50 μm). *p* < 0.05, values expressed as mean ± SD (*n* = 7), * significant vs. CON group, # significant vs. DOX group, & significant vs. DCN-L+DOX group. CON: control; DOX: doxorubicin; DCN-L: diacerein low dose; DCN-H: diacerein high dose.

Masson’s trichrome-stained tissue sections of the DOX group delineated a high concentration of collagen fiber deposition in between the cardiomyocytes. In comparison, DCN-L+DOX and DCN-H+DOX demonstrated moderate and scanty collagen fibers between cardiomyocytes. Quantitative analysis of MASSON-stained heart sections revealed a significant decrease in collagen fiber distribution among cardiomyocytes in the DCN-L+DOX and DCN-H+DOX groups compared to the DOX group, in which group DCN-H+DOX demonstrated the best improvement. The treated groups showed noticeable improvement but did not reach the control level (Figure 4).

### 3.4. DCN Effect on Cardiac ATF3, NCOA4-Mediated Ferritinophagy, and Ferrous Iron Content

**Figure 5:**
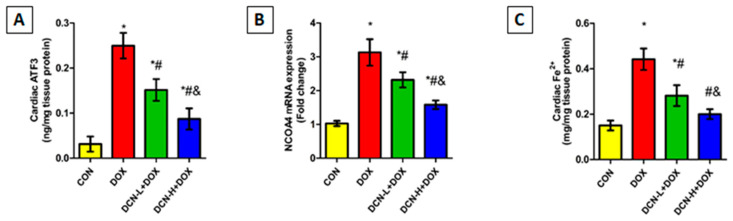
Cardiac ATF3, NCOA4-mediated ferritinophagy, and ferrous iron content among different studied groups. (**A**) Cardiac ATF3 (ng/mg tissue protein). (**B**) NCOA4 relative gene expression. (**C**) Cardiac Fe^2+^ (mg/mg tissue protein). CON, control; DOX, doxorubicin; DCN-L, diacerein low dose; DCN-H, diacerein high dose; ATF3, activating transcription factor 3; NCOA4, nuclear receptor co-activator 4. *p* < 0.05, values expressed as mean ± SD (*n* = 7), * significant vs. CON group, # significant vs. DOX group, & significant vs. DCN-L+DOX group.

As illustrated in Figure 5, cardiac ATF3 levels were substantially higher in the DOX-intoxicated group coupled with upregulated NCOA4 expression compared to those of the CON group. Meanwhile, DCN co-administration effectively diminished the escalation in ATF3 levels and downregulated NCOA4 relative expression compared to the DOX group, with a better impact in the high-dose group.

Considering the role of cellular iron overload in ferroptosis, heart ferrous iron content was assessed. As depicted in Figure 5, there was a substantial rise in cardiac Fe^2+^ following i.p. injection of DOX in contrast to the CON group. Conversely, the DCN-treated groups showed a considerable decline in cardiac Fe^2+^, with more pronounced results in the high-dose group.

### 3.5. DCN Abolished the DOX-Provoked Cardiac OS and Inflammation

DOX-injected rats displayed a marked rise in cardiac MDA and 4-HNE levels, which indicates iron-based lipid peroxidation concomitant with lower GSH levels than the CON group. These results shown in Table 2 suggest DOX-triggered ferroptosis. Additionally, the level of the oxidative DNA damage biomarker 8-OHdG was greatly enhanced following DOX injection compared to the CON group. Meanwhile, DCN administration reversed the above-mentioned parameters in a dose-dependent manner. These findings suggest that DCN treatment successfully mitigates ferroptosis and OS elicited by DOX treatment in a dose-dependent manner.

Concerning cardiac inflammatory biomarkers, DOX administration evoked myocardial inflammation, as revealed by substantial elevations in IFN-γ, IL-6, and HMGB1 levels compared to the CON group. Interestingly, DCN exhibited a potent anti-inflammatory effect, as indicated by a notable reduction in the above-mentioned parameters in DCN-administered rats in comparison to the untreated DOX group. This reduction was more prominent at high DCN doses. Even so, it is still significantly higher than the CON group (Table 2).

### 3.6. DCN-Abolished DOX-Aggravated Phospho-P53 Immunoreactivity in Rats’ Cardiac Tissue

**Figure 6:**
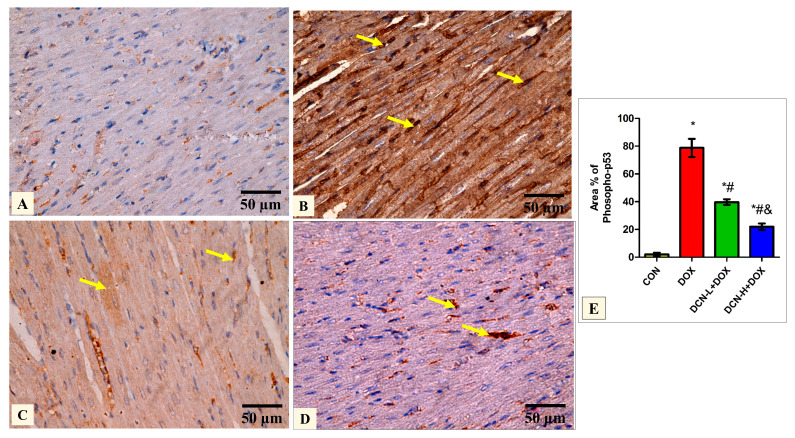
Modulatory effect of DCN on phospho-p53 immunohistochemical expression in heart tissue. (**A**) The CON group demonstrates a negative phospho-p53 immunoreaction. (**B**) The DOX group displays a strong positive phospho-p53 reaction in most cardiomyocytes’ nuclei (yellow arrows). (**C**) The DCN-L+DOX group demonstrates a moderate phospho-p53-positive reaction as a faint brown color in some cardiomyocytes. (**D**) The DCN-H+DOX group exhibits a weak phospho-p53-positive reaction in a few cardiomyocytes’ nuclei (arrows). (**E**) Area percentage of phosphor-p53 in all groups. (phospho-p53 immunostaining ×400, scale bar = 50 m). *p* < 0.05, values are expressed as mean ± SD (*n* = 7), * significant vs. CON group, # significant vs. DOX group, & significant vs. DCN-L+DOX group. CON, control; DOX, doxorubicin; DCN-L, diacerein low dose; DCN-H, diacerein high dose.

Quantitative analysis of the mean percentage of phospho-p53 revealed that the treated groups DCN-L+DOX and DCN-H+DOX showed a notable decline compared to the diseased DOX group, with the DCN-H+DOX having the greatest improvement. There was a noticeable improvement in the treated groups; however, they did not reach the level of the CON group (Figure 6).

### 3.7. DCN Reinforced the Cardiomyocytes’ Resistance to Ferroptosis by Activating the NRF2 Signaling

**Figure 7:**
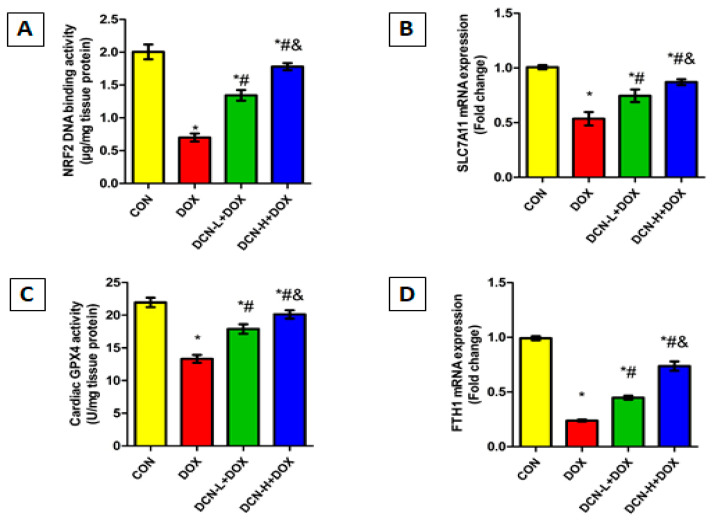
DCN enhanced NRF2 signaling in rats’ cardiac tissue. (**A**) Cardiac NRF2 DNA-binding activity (µg/mg tissue protein). (**B**) Relative SLC7A11 mRNA expression. (**C**) Cardiac GPX4 activity (U/mg tissue protein). (**D**) Relative FTH1 mRNA expression. CON, control; DOX, doxorubicin; DCN-L, diacerein low dose; DCN-H, diacerein high dose; NRF2, nuclear factor erythroid 2–related factor 2; SLC7A11, solute carrier family 7 member 11; GPX4, glutathione peroxidase 4; FTH1, ferritin heavy chain 1. *p* < 0.05, values are expressed as mean ± SD (*n* = 7), * significant vs. CON group, # significant vs. DOX group, & significant vs. DCN-L+DOX group.

Considering the crucial role of NRF2 in maintaining cellular redox homeostasis and regulating transcription of various genes related to iron storage (FTH1) and ferroptosis (SLC7A11 and GPX4), we speculate that upregulating NRF2 could be effective in guarding against DOX-evoked ferroptosis. So, we assessed the NRF2 DNA-binding activity, FTH1 and SLC7A11 mRNA relative expressions, and the potent anti-peroxidation enzyme GPX4 in the cardiomyocytes of all tested groups. DOX administration dramatically declined NRF2 DNA binding activity, downregulated FTH1 and SLC7A11 relative expression, and suppressed GPX4 catalytic activity, predisposing cardiomyocytes to ferroptotic cell death. By reversing the aforementioned parameters, DCN exhibited an anti-ferroptosis effect, protecting cardiomyocytes from DOX-triggered ferroptosis in a dose-dependent manner (Figure 7).

### 3.8. DCN Mitigated DOX-Evoked Cardiac ER Stress and Apoptosis

**Figure 8:**
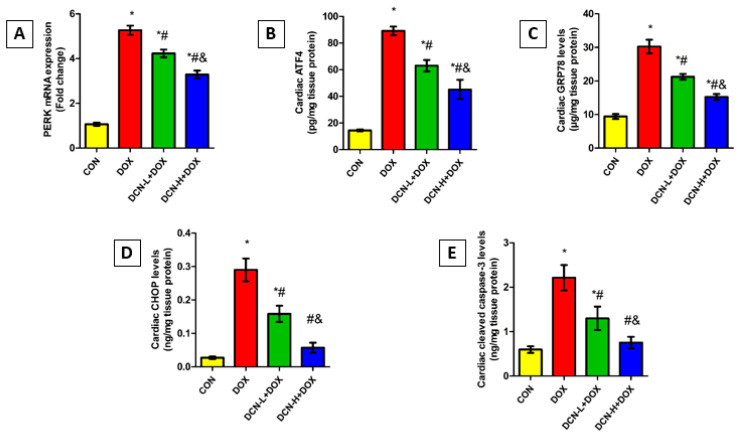
Modulatory effect of DCN on cardiac ER stress and apoptotic markers. (**A**) Relative PERK mRNA expression. (**B**) Cardiac ATF4 (pg/mg tissue protein). (**C**) Cardiac GRP78 levels (μg/mg tissue protein). (**D**) Cardiac CHOP levels (ng/mg tissue protein). (**E**) Cardiac cleaved caspase-3 levels (ng/mg tissue protein). CON, control; DOX, doxorubicin; DCN-L, diacerein low dose; DCN-H, diacerein high dose; PERK, protein kinase R-like ER kinase; ATF4, activating transcription factor 4; GRP78, glucose-regulated protein 78; CHOP, C/EBP homologous protein. *p* < 0.05, values are expressed as mean ± SD (*n* = 7), * significant vs. CON group, # significant vs. DOX group, & significant vs. DCN-L+DOX group.

As shown in Figure 8, the DOX-intoxicated rats showed a notably upregulated PERK relative expression concomitant with elevated ATF4, GRP78, and CHOP levels relative to the CON group. Remarkably, DCN treatment abolished DOX-elicited ER stress, denoted by an observable reduction in the above-mentioned parameters in a dose-dependent manner. Concurrently, cleaved caspase-3 levels were considerably higher in the DOX group than in the CON one. Meanwhile, treatment with DCN effectively protected cardiomyocytes from DOX-aggravated apoptosis, as indicated by substantially reduced cardiac cleaved caspase-3, suggesting a potential anti-apoptotic effect of DCN.

### 3.9. Correlation between Cardiac NRF2 and Other Measured Parameters in the DCN High Dose-Treated Group

**Figure 9:**
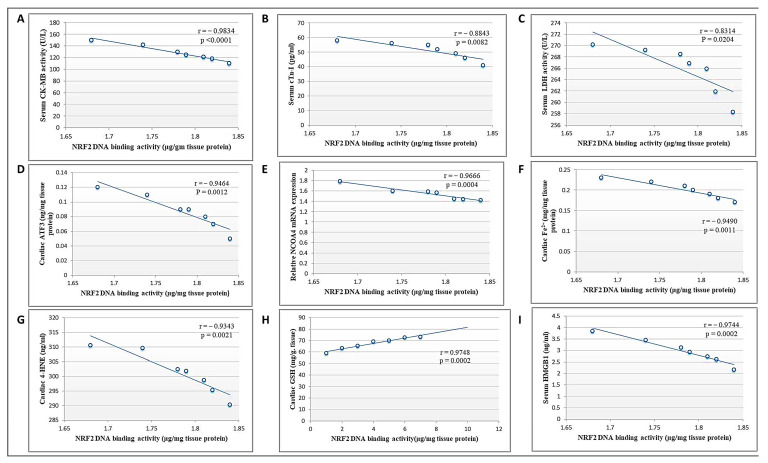
Correlation between cardiac NRF2 and other measured parameters in the DCN high-dose-treated group. (**A**) CK-MB activity (U/L). (**B**) cTn-I levels (pg/mL). (**C**) LDH activity (U/L). (**D**) ATF3 level (ng/mg protein). (**E**) NCOA4 mRNA level. (**F**) Fe^2+^ (mg/mg protein). (**G**) 4-HNE (ng/g tissue). (**H**) GSH content (mg/g tissue). (**I**) HMGB1 (ng/mL) in the high-dose DCN-treated group.

Pearson’s correlation analysis showed that NRF2 DNA-binding activity was negatively correlated with cardiac toxicity indices (CK-MB, cTn-I, and LDH), cardiac ATF3, NCOA4 mRNA expression, Fe^2+^, and 4-HNE levels, as well as serum HMGB1 levels. Meanwhile, there was a positive correlation between DNA binding activity and cardiac GSH (Figure 9). Collectively, we suggest that DCN attenuated cardiac damage and counteracted DOX-provoked ferroptosis via upregulating NRF2.

## 4. Discussion

The current work is the first to emphasize the prospect of DCN as a cardioprotective pharmaceutical agent and probe the likely signaling cascades engaged in its shielding potential. Our results suggested that DCN prevented DIC in a tangible way, as it was proven to correct the deranged cardiac injury indices, the reported cytomorphological alterations, and the distorted HW/BW ratio in a dose-dependent manner. Such demonstrations supported the DCN cardioprotective potential suggested in preceding works [27,28,29].

The present work verified that ferroptosis was a fundamental hallmark and cornerstone of DIC pathogenesis, as previously reported [30,31]. Following Li et al. [30], our data revealed a pronounced cardiac ferrous iron increment. In line with Hanna et al. [31], our study highly recommended NCOA4-mediated ferritinophagy as an upper-hand mechanism beyond the manifest iron overload. Further analysis pointed to the IL-6-dependent inhibition of iron efflux via ferroportin as a cardinal contributor [32,33]. Moreover, consistent with previous studies [34,35,36,37], our data evidenced that ER stress-dependent ATF3-mediated transferrin receptor-1 (TFR1) upregulation was a hidden hand behind the expanded cardiac labile iron pool (LIP).

In line with earlier reports, we denoted the significant downregulation of cardiac FTH1 and SLC7A11 in the DOX-intoxicated rats [30,31]. Such findings definitely contributed to cardiac LIP expansion. For SLC7A11 downregulation, numerous rationales were stated herein. The first was the ATF3-mediated downregulation of SLC7A11 mRNA expression, as formerly validated [38]. The second involved p53, a cardinal DIC transcriptomic regulator [39]. Mechanistically, the DOX-induced DNA damage motivated p53 phosphorylation, promoting its dissociation from the mouse double minute 2 homolog, its nuclear translocation, and DNA binding to ultimately inhibit SLC7A11 transcription [40]. Lastly, the augmented cardiac IFN-γ could participate via interferon-regulatory factor 1 upregulation, as reported by Zeng et al. [41].

Fortunately, as a study novelty, our data revealed that DCN effectively and dose-dependently hindered the cardiomyocytes’ ferroptotic cell death signal, insinuated by the attenuated serum HMGB1 and the recorded architectural ameliorations. A two-armed mechanism involving obstruction of cardiac LIP expansion and lipid peroxide accumulation was included in the DCN anti-ferroptotic effect.

Being a member of the anthraquinone family could partly illustrate the exhibited DCN’s anti-ferroptotic impact. In fact, DCN is a di-acetylated derivative of the anthraquinone rhein, which is actually the bioactive metabolite of DCN [42]. Rhein, as a phenolic compound, exerts a direct ROS-scavenging effect that was literally explained on three different bases. The first one is the hydrogen atom transfer mechanism. The second mechanism is single-electron transfer followed by proton transfer, while the third is sequential proton loss and electron transfer. This is of great significance in attenuating cardiac lipid peroxide accumulation and maintaining cardiac redox homeostasis [43].

In attempts to dissect more in-depth molecular mechanisms, our analyses and correlation studies indicated that DCN’s anti-ferroptotic signature relied upon NRF2 upregulation. Physiologically, NRF2 is sequestered in the cytosol and bound to KEAP-1. KEAP-1 is an adaptor of the Cullin3-based ubiquitin E3 ligase that enhances NRF2 ubiquitination and proteasomal degradation [44]. Interestingly, rhein was reported to orient itself at the site of KEAP-1, inhibiting the KEAP-1-NRF2 interaction, increasing the NRF2 cytosolic pool, and allowing its nuclear translocation to regulate its target gene expression [45]. Moreover, the DCN-induced NRF2 upregulation could be related to the unique interplay between NRF2 and p53. It is worth noting that p53, when relatively low, as evident in the DCN groups, enhances transcription of NRF2 alongside its target genes [46]. Additionally, owing to the anthraquinone’s epigenetic-modifying nature [47], DCN could exert a direct stimulatory effect on NRF2 transcriptional machinery. However, further studies are warranted to confirm such a perspective.

In the same framework, our data put forward numerous hypotheses to illustrate the DCN-induced reduction in cardiac ferrous iron; all appeared to be related to the boosted NRF2 level. The first was the dose-dependent attenuation of NCOA4-related ferritinophagy, mostly controlled by NRF2, as recommended by Anandhan et al. [48]. The second was the enhancement of ferroportin-mediated iron export because of the IL-6 transcriptional blockade identified here and supported previously. The reported IL-6 transcriptional blockade could be attributed to the direct redox-related signature of DCN and/or NRF2 imprint, besides the well-documented DCN’s anti-inflammatory potential, as acknowledged previously [11,19,49]. Interference with TFR1-mediated iron import constituted a third motion based on DCN’s ability to abrogate the provoked ER stress signal. The reported ER stress-relieving impact of DCN aligned with numerous reports [11,12,14], which attributed that directly to the modulatory effect of DCN on ER calcium content and numerous ER stress-related biomolecules and indirectly to the NRF2 antioxidant effect. Highlighting the NRF2 imprint in the DCN’s anti-ferroptotic impact, the DCN-treated animals exhibited SCL7A11 and FTH1 upregulation, reinforcing resistance to ferroptosis. Both genes are direct NRF2 transcriptional targets. Thus, our findings were reliable considering the recorded NRF2 upregulation, as affirmed previously [11,19,30,50]. SLC7A11 upregulation was obviously reflected in the catalytic activity of GPX4, the cardinal ferroptotic inhibitor [9].

**Figure 10:**
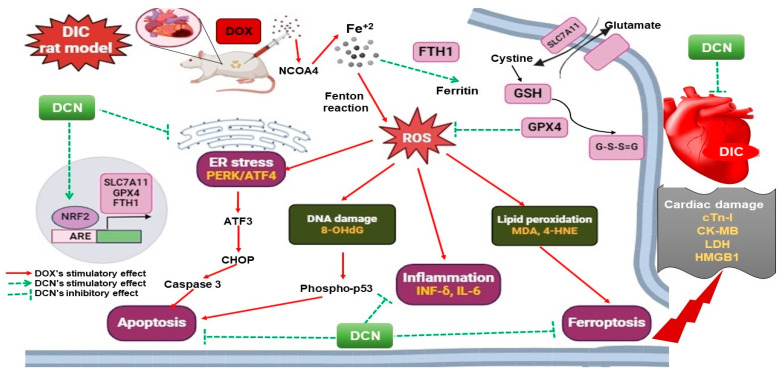
The proposed mechanisms implicated in DCN’s cardioprotective potency against DIC in rats.

Lastly, we scrutinized that apoptosis was the convergence point of the distinct DIC precipitating signaling cascades, where it was denoted by an enhancement of the cardiac caspase-3 level in the DOX group. In line with prior works, numerous speculations were imposed herein, including the DOX-driven OS, p53 activation, and the ER stress-induced CHOP transcription [51,52,53,54]. Within expectations and in line with a published work [11], DCN was proven to alleviate the apoptotic cell death signal valuably and dose-dependently. Literally, this was attributed to the DCN’s antioxidant, anti-inflammatory, and ER stress-relieving effects. A summary of the mechanisms that explain DCN’s ability to mitigate DOX cardiac damage to rats’ hearts is illustrated in Figure 10.

The principal study limitation is the lack of the DCN-only group to confirm any potential adverse effects and their impact on the parameters under investigation. Another potential limitation is the lack of functional assessments.

## 5. Conclusions

The present work acknowledges for the first time the cardioprotective potency of DCN against DIC. To the best of our knowledge, this is the first work scrutinizing DCN’s anti-ferroptotic signature, where our data emphasized that rats’ pretreatment with DCN effectively and dose-dependently reinforced the cardiomyocytes’ resistance to ferroptosis mainly by restoring the NRF2/SLC7A11/GPX4 signaling axis. Combating the DOX-induced LIP expansion alongside its consequent lethal lipid peroxide overproduction was a secret code promoting the DCN anti-ferroptotic imprint. Over and above, DCN valuably abrogated the cardiomyocytes’ suicide signal that appeared herein as a common final step and a significant convergence point of all the DIC precipitating pathways. From our point of view, DCN constituted a promising therapeutic avenue and represented a glimmer of hope for those under DOX-dependent chemotherapeutic protocols, being able to circumvent such life-threatening side effects.

## Figures and Tables

**Table 1:** Primer sequences.

Gene	Accession Number	Forward (5′-3′)	Reverse (5′-3′)
NCOA4	NM_001034007.1	TGAAGTGCAGTGCTCACACA	TTCGCTGCTGCTGACAGTTA
FTH1	NM_012848.2	CCCTTTGCAACTTCGTCGCT	CTCCGAGTCCTGGTGGTAGT
SLC7A11	NM_001107673.3	GAGGCGCTGTAGCCACATTA	GGCATTCAACCAGGTGATCC
PERK	NM_031599.2	GAAGTCGAGAGGCGTGGGGG	GCCCGTCTCGCCGCTAGGAG
GAPDH	NM_017008.4	GGCATCGTGGAAGGGCTC	GACCTTGCCCACAGCCTT

**Table 2:** Modulatory effect of DCN against DOX-induced OS and inflammation in rat’s cardiac tissue.

Parameter/Groups	CON	DOX	DCN-L+DOX	DCN-H+DOX
Cardiac GSH (mg/g tissue)	86.29 ± 4.09	25.27 ± 3.11 *	47.86 ± 4.43 *#	67.54 ± 5.23 *#&
Cardiac MDA (nmol/g. tissue)	5.043 ± 1.54	19.89 ± 1.10 *	16.09 ± 1.14 *#	12.64 ± 1.83 *#&
Cardiac 4-HNE (pg/g tissue)	114.9 ± 6.42	566.2 ± 9.94 *	430.0 ± 9.92 *#	301.3 ± 7.33 *#&
Cardiac 8-OHdG (ng/g tissue)	4.614 ± 1.20	41.00 ± 4.93 *	30.64 ± 4.11 *#	14.20± 4.13 *#&
Cardiac IL-6 (pg/mg tissue)	5.18 ± 0.97	31.81 ± 3.26 *	25.09 ± 1.24 *#	20.46 ± 1.25 *#&
Cardiac IFN-γ (pg/mg tissue)	25.13 ± 3.56	97.66 ± 1.69 *	58.17 ± 3.70 *#	38.81 ± 5.69 *#&
Serum HMGB1 (ng/mL)	1.821 ± 0.103	7.587 ± 0.73 *	4.764 ± 0.61 *#	2.983 ± 0.55 *#&

Significant at a *p*-value < 0.05; values are expressed as mean ± SD, 7 rats of each group, * significant vs. CON group, # significant vs. DOX group, & significant vs. DCN-L+DOX group. CON, control; DOX, doxorubicin; DCN-L, diacerein low dose; DCN-H, diacerein high dose; GSH, reduced glutathione; MDA, malondialdehyde; 4-HNE, 4-hydroxynonenal; 8-OHdG, 8-hydroxy-2′-deoxyguanosine; IL-6, interleukin 6; IFN-γ, interferon-gamma; HMGB1, high mobility group box 1 protein.

## Data Availability

The corresponding authors can provide the data used to verify the findings of this research upon request.
